# Prevalence of equine lungworm (*Dictyocaulus arnfieldi*) infection and associated risk factors in Egypt

**DOI:** 10.3389/fvets.2026.1895496

**Published:** 2026-07-17

**Authors:** Hattan S. Gattan, Mohamed Marzok, Mohammed H. Alruhaili, Hesham Ismail, Adel I. Almubarak, Abdelfattah Selim

**Affiliations:** 1 Department of Medical Laboratory Sciences, Faculty of Applied Medical Sciences, King Abdulaziz University, Jeddah, Saudi Arabia; 2 Special Infectious Agents Unit, King Fahad Medical Research Center, King AbdulAziz University, Jeddah, Saudi Arabia; 3 Department of Clinical Sciences, College of Veterinary Medicine, King Faisal University, Al-Ahsa, Saudi Arabia; 4 Department of Clinical Microbiology and Immunology, Faculty of Medicine, King AbdulAziz University, Jeddah, Saudi Arabia; 5 Department of Public Health, College of Veterinary Medicine, King Faisal University, Al-Ahsa, Saudi Arabia; 6 Department of Animal Medicine (Infectious Diseases), Faculty of Veterinary Medicine, Benha University, Toukh, Egypt

**Keywords:** *Dictyocaulus arnfieldi*, Egypt, equine, prevalence, risk factors

## Abstract

**Introduction:**

*Dictyocaulus arnfieldi* infection is a common parasitic respiratory disease of equines that can adversely affect animal health, welfare, and productivity. However, epidemiological information on this infection remains limited in many regions of Egypt. This study aimed to determine the prevalence of *D. arnfieldi infection* and identify associated risk factors among equines in three Egyptian governorates.

**Methods:**

A cross-sectional study was conducted on 415 equines, including 150 donkeys, 55 mules, and 210 horses. Animals were examined for *D. arnfieldi infection*, and data on species, age, body condition, and deworming history were collected. Associations between infection and potential risk factors were evaluated using appropriate statistical analyses.

**Results:**

The overall prevalence of *D. arnfieldi* infection was 41% (170/415). Donkeys had the highest prevalence (51.3%), whereas mules showed the lowest prevalence (20%). Significant associations (*p* < 0.05) were observed between infection and age, body condition, and deworming history. Equines older than 8 years exhibited the highest prevalence (54.7%), followed by animals with poor body condition (52.4%) and those with no history of deworming (54.6%).

**Discussion:**

The high prevalence of D. arnfieldi, particularly among donkeys, confirms their important role as the primary reservoir host, contributing to sustained transmission among equine populations. These findings highlight the need for targeted parasite control strategies, including routine deworming, improved veterinary care, and better management practices, especially for donkeys, to reduce lungworm infection and improve equine health and productivity.

## 1 Introduction

Donkeys, mules, and horses play important roles as working animals in Egypt, particularly in transportation and agricultural activities. Maintaining their health and productivity is therefore essential. However, parasitic diseases, including lungworm infection caused by *Dictyocaulus arnfieldi*, can adversely affect respiratory health, welfare, and work performance, highlighting the need for epidemiological studies and effective control measure (1).

Parasitic infections represent a significant cause of morbidity in equines, compromising their health and productivity in many regions of the world (2). A wide range of parasites has been associated with respiratory disorders in these animals, with several studies reporting their clinical and epidemiological importance. The main respiratory manifestations associated with *D. arnfieldi* infection, such as coughing, nasal discharge, increased respiratory rate, dyspnea, reduced exercise tolerance, and general weakness, particularly in heavily infected animals (3, 4). Among these parasites, species of the genus *Dictyocaulus* are recognized as the most important contributors to respiratory disease. This is largely attributed to the fact that equines serve as natural reservoirs for the parasite, combined with its widespread and ubiquitous distribution in diverse ecological environments (5–7).

*D. arnfieldi* is the primary lungworm species affecting equines, including donkeys, horses, ponies, and zebras, and is distributed worldwide (8). Donkeys and their hybrids, such as mules, serve as the natural hosts for this parasite, and infections in horses are most commonly observed when they are kept in close contact with donkeys or mules (9, 10). The adult worms are slender, medium-sized nematodes, typically whitish to grayish in appearance. Morphologically, *D. arnfieldi* possesses both a digestive and a nervous system; however, it lacks a distinct excretory system (11, 12).

Equines acquire lungworm infection primarily through grazing on contaminated pastures; however, transmission can also occur indoors via infected hay or bedding materials (13, 14). The pathological changes associated with lungworm infection in equines are typically categorized into three phases: prepatent, patent, and post-patent. The severity and manifestation of these pathological effects are influenced by several factors, including the specific location of the parasites within the respiratory tract, the number of infective larvae ingested, and the host’s immune competence. Additionally, the body condition, nutritional status, and age of the animal play crucial roles in determining susceptibility and disease outcome (15, 16).

The prevalence of *D. arnfieldi* infection in equines varies considerably across different regions, ranging from low to high levels depending on management and environmental conditions. Studies have consistently reported a higher prevalence in donkeys than in horses and mules, as donkeys act as the principal reservoir host and often remain asymptomatic while shedding infective larvae. Several risk factors have been identified, including species, age, body condition, grazing practices, and deworming history. Equines kept under extensive grazing systems, those with poor body condition, older animals, and those lacking regular anthelmintic treatment show significantly higher infection rates. Mixed grazing of horses with donkeys has also been recognized as an important factor facilitating parasite transmission and maintaining infection within equine populations (17, 18).

Despite the large equine population in Egypt, research on equine lungworm remains scarce. Furthermore, potential risk factors such as age, body condition, and sex, and their association with lungworm infection in equines, have not been adequately investigated.

Therefore, the present study was undertaken to determine the prevalence of lungworm infection among equines in three Egyptian governorates and to evaluate the related risk factors contributing to dictyocaulosis in this population.

## 2 Materials and methods

### 2.1 Ethical statement

The study was conducted in accordance with the guidelines of the Ethical Committee of the Faculty of Veterinary Medicine, Benha University, and received formal approval (Approval Nr: FVTM02-04-2023) from the committee. Informed consent was obtained from all animal owners prior to the commencement of the experimental procedures, ensuring their understanding and agreement to participate in the study. Furthermore, all procedures were carried out in compliance with the ARRIVE guidelines.

### 2.2 Study area

**Figure 1:**
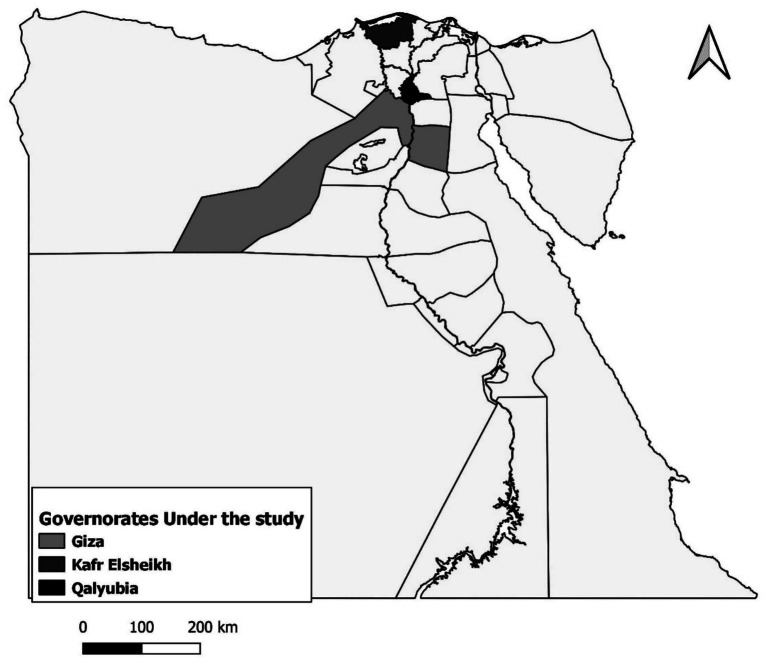
MAP showed governorates under the study (MAP generated by QGIS software).

The study was carried out between January and December 2023 across three Egyptian governorates: Giza, Kafr El-Sheikh, and Qalyubia (Figure 1).

The three governorates under study including Giza, Kafr El Sheikh, and Qalyubia—are located in the northern part of Egypt and share a predominantly subtropical to arid climate. Giza is characterized by hot, dry summers and mild winters with low rainfall, while Kafr El-Sheikh, being part of the Nile Delta, experiences relatively higher humidity and moderate temperatures due to its proximity to the Mediterranean Sea. Qalyubia, situated in the central Nile Delta, has a climate intermediate between the two, with warm summers and mild winters, accompanied by moderate humidity levels. These climatic variations can influence the prevalence of lungworm infection in horses.

### 2.3 Sampling and sample size

The present investigation was designed as a cross-sectional study, aiming to estimate the prevalence of lungworm infection and assess associated risk factors in equines. A simple random sampling technique was employed to ensure that each individual animal within the study population had an equal chance of being selected, thereby minimizing selection bias and enhancing the representativeness of the sample. The required sample size was determined using the standard formula described by Thrusfield (19). In this calculation, an expected prevalence of 50% was assumed in order to maximize the sample size and achieve the highest precision. The confidence level was set at 95%, and the margin of error was maintained at 5%, ensuring reliable statistical power. Based on this calculation, the minimum sample size required for the current study was estimated to be 384 equines.

### 2.4 Fecal examination

Fecal samples were obtained directly from the rectum of each animal using sterile gloves to avoid contamination. The samples were then transferred into appropriately labeled vials and immediately transported to the Veterinary Diagnostic Laboratory, Faculty of Veterinary Medicine, Benha University. During sampling in the field, all fecal samples were clearly labeled with the date of sampling, the origin, and the animal species. All fecal samples were processed on the same day of collection to ensure sample integrity. Parasitological examination was carried out using the modified Baermann technique, as described by Foreyt (20). The recovered larvae of *Dictyocaulus arnfieldi* were identified based on their morphological characteristics, including body length, shape of the anterior end, structure of the esophagus, and tail morphology, in accordance with the available identification keys (21). In addition, the number of larvae per 1 g of faeces (LPG) was determined according to study carried out by Thienpont et al. (46), Kiszely et al. (47) and the level of infection was determined according to Seyoum et al. (22) as none, mild <500 egg per gram (EPG), moderate (500–1,000 EPG) and high (>1,000 EPG).

### 2.5 Questionnaire

During the sampling process, a structured questionnaire was used to record potential risk factors associated with lungworm infection. Information was collected on several variables, including the locality, sex (male or female), age category, species of the equine (donkey, horse, or mule), body condition score (poor, medium, or good), and deworming history (whether the animal had previously received anthelmintic treatment or not). Assessing Body Condition Score (BCS) in horses involves evaluating fat cover and muscle tone at specific anatomical sites. The most widely used system is the Henneke 9-point scale (23), but simplified 3-category scoring (poor, medium, good). These data were gathered directly from animal owners and were systematically documented for subsequent analysis of their association with lungworm prevalence.

### 2.6 Data analysis

The collected data were systematically recorded on pre-designed data collection forms and subsequently entered into Microsoft Excel for preliminary organization and cleaning. The primary outcome variable was the detection of lungworm *D. arnfieldi* larvae during fecal examination, categorized as positive (1) or negative (0). Descriptive statistical analysis were performed to determine the overall prevalence of lungworm infection and to calculate the proportion of infected animals within each equine species.

To further explore potential associations, logistic regression analysis was applied to evaluate the relationship between identified risk factors and the presence of infection. For each variable, odds ratios (ORs) were computed to measure the strength and direction of the association. Statistical significance was assessed at a 95% confidence interval (CI) with a *p*-value of less than 0.05 considered significant. All statistical analyses were conducted using SPSS software, version 24 (IBM, USA).

## 3 Results

Out of a total of 415 examined equines, 170 animals tested positive for lungworm infection, corresponding to an overall prevalence of 41%. When analyzed by species, the prevalence from the positive samples was 51.3% in donkeys, 20% in mules, and 39% in horses. Statistical analysis, however, revealed no significant association between animal species and prevalence of infection (*p* > 0.05). 82 Out of 210 infected horses, 33 (15.7%) were affected mildly,26 (12.4%) moderately and 22 (10.5%) highly.

The intensity of infection showed a clear relationship with the health status of the examined animals. Higher infection intensities were observed in animals with poor body condition.

With respect to the locality and the sex, no statistically significant differences were observed in the prevalence of lungworm infection. The highest prevalence by locality was recorded in Kafr El-sheikh (45.7%), while by sex, females (42.3%) showed a slightly higher prevalence compared to males (Table 1).

**Table 1:** Prevalence of lungworm infection in equine in relation to factors studied.

Variable	No of examined animals	No of positive	% of positive	95% CI	Statistic
Locality					
Giza	120	51	42.5	34.02–51.44	χ^2^ = 3.348, df = 2, *p* = 0.187
Kafr El-Sheikh	140	64	45.7	37.68–53.97	
Qalyubia	155	55	35.5	28.38–43.28	
Sex					
Male	110	41	37.3	28.81–46.59	χ^2^ = 0.843, df = 1, *p* = 0.358
Female	305	129	42.3	36.89–47.91	
Age					
<3 years	143	41	28.7	21.89–36.56	χ^2^ = 19.811, df = 2, *P* < 0.0001***
3–8 years	133	53	39.8	31.93–48.34	
>8 years	139	76	54.7	46.39–62.72	
Species					
Mule	55	11	20.0	11.55–32.36	χ^2^ = 16.983, df = 2, *p* < 0.0001***
Horse	210	82	39.0	32.7–45.79	
Donkey	150	77	51.3	43.4–59.2	
Seasons					
Winter	125	55	52.4	44.35–60.29	χ^2^ = 0.850 df = 3 *p* = 0.838
Spring	130	53	40.8	32.71–49.36	
Summer	110	42	38.2	29.65–47.51	
Autumn	50	20	40	27.61–53.82	
Body condition					
Poor	147	77	52.4	44.35–60.29	χ^2^ = 13.908, df = 2, *p* = 0.001**
Medium	135	52	38.5	30.74–46.94	
Good	133	41	30.8	23.61–39.12	
Deworming history					
Dewormed	155	28	18.1	12.8–24.86	χ^2^ = 53.646, df = 2, *P* < 0.0001***
Non-dewormed	260	142	54.6	48.54–60.56	
Total	415	170	41.0	36.33–45.75	

*Results are significant if *p* < 0.05.

The prevalence of lungworm infection was varied seasonally (*p* = 0.838), with higher infection rates recorded during winter (52.4%), while the lower value were observed during summer season (38.2%).

The prevalence of lungworm infection was significantly associated with age, species, body condition, and deworming history. Higher infection rates were recorded in animals over 8 years of age (54.7%), donkeys (51.3%), animals with poor body condition (52.4%), and those without a history of deworming treatment (54.6%) (Table 1).

The prevalence of lungworm infection was significantly associated with age, species, body condition, and deworming history (Table 1). The highest prevalence was observed in animals older than 8 years (54.7%), followed by donkeys (51.3%), animals with poor body condition (52.4%), and animals with no history of deworming (54.6%). These associations were statistically significant (*p* < 0.05).

The multivariable logistic regression analysis revealed a significant association between lungworm infection and several risk factors, including age, species, body condition, and deworming history (*p* < 0.05). Animals over 8 years of age were more likely to be infected (OR = 2.7; 95% CI: 1.57–4.58) compared to those younger than 3 years. Donkeys also showed a significantly higher likelihood of infection (OR = 2.8; 95% CI: 1.24–6.21) compared to other equine species. Similarly, animals with poor body condition were at greater risk (OR = 2.2; 95% CI: 1.30–3.86) than those with good body condition. The strongest predictor was deworming history, as non-dewormed animals were substantially more likely to be infected (OR = 4.6; 95% CI: 2.80–7.59) compared to those that had received deworming treatment, Table 2.

**Table 2:** Multivariate logistic regression analysis for risk factors related to lungworm infection in equine.

Variables	B	S.E.	OR	95% CI for OR		*p* value
				Lower	Upper	
Age						
<3 years	Ref.					
3–8 years	0.474	0.280	1.6	0.93	2.78	0.091
>8 years	0.984	0.274	2.7	1.57	4.58	0.000
Species						
Mule	Ref.					
Horse	0.572	0.400	1.8	0.81	3.89	0.153
Donkey	1.019	0.412	2.8	1.24	6.21	0.013
Body condition						
Good	Ref.					
Poor	0.809	0.277	2.2	1.30	3.86	0.003
Medium	0.189	0.282	1.2	0.69	2.10	0.503
Deworming history						
Dewormed	Ref.					
Non-dewormed	1.528	0.255	4.6	2.80	7.59	0.000

B, Logistic regression coefficient; SE, Standard error, OR, Odds ratio, 95%CI, Confidence interval.

Table 3 presents the prevalence of lungworm infection according to various risk factors in equines. The findings revealed higher infection rates among older animals and females, with the greatest prevalence observed in individuals with poor body condition across all examined equine species.

**Table 3:** Prevalence of lungworm in association with risk factors of equines.

Variables	Giza		Kafr El-Sheikh		Qalyubia		Total	
	Examined	Infected (%)	Examined	Infected (%)	Examined	Infected (%)	Examined	Infected (%)
Horse								
Age								
<3 years	12	2 (16.7)	12	2 (16.7)	12	2 (16.7)	36	6 (16.7)
3–8 years	31	8 (25.8)	27	8 (29.6)	20	8 (40)	78	24(30.8)
>8 years	37	20 (54.1)	31	23 (74.2)	28	20 (71.4)	96	53 (65.6)
Sex								
Male	35	9 (25.7)	23	6 (26.1)	17	5 (29.4)	75	20 (26.7)
Female	75	31 (41.3)	43	20 (46.5)	17	11 (64.7)	135	62 (46)
Body condition								
Poor	32	18 (56.3)	22	15 (68.2)	19	12 (63.2)	73	45 (61.7)
Medium	25	12 (48)	21	9 (42.8)	18	8 (44.4)	64	28 (43.8)
Good	36	4 (11.1)	24	3 (12.5)	13	2 (15.4)	73	9 (13.3)
Donkey								
Age								
<3 years	5	2 (40)	8	2 (25)	8	1 (12.5)	21	7 (23.8)
3–8 years	7	6 (85.7)	22	10 (45.5)	26	12 (46.2)	55	28 (51)
>8 years	8	7 (87.5)	31	13 (42)	35	12 (34.3)	74	42 (43.2)
Sex								
Male	9	2 (22.2)	14	8 (57.1)	11	5 (45.5)	34	15 (44.1)
Female	17	15 (88.2)	66	28 (42.4)	33	19 (57.6)	116	62 (53.4)
Body condition								
Poor	12	8 (66.6)	24	20 (83.3)	12	9 (75)	48	37 (77.1)
Medium	9	7 (77.8)	26	13 (50)	13	9 (69.2)	48	29 (60.4)
Good	11	2 (18.2)	28	6 (21.4)	15	3 (20)	54	11 (20.4)
Mules								
Age								
<3 years	2	1 (50)	1	0	2	0	5	1 (20)
3–8 years	6	2 (33.3)	3	2 (66.7)	12	1 (8.3)	21	4 (23.8)
>8 years	12	3 (25)	5	3 (60)	12	2 (16.7)	29	6 (27.6)
Sex								
Male	1	0	1	0	1	0	2	0
Female	1	0	22	4 (18.2)	30	7 (23.3)	53	11 (20.7)
Body condition								
Poor	3	1 (33.3)	10	3 (30)	9	2 (22.2)	22	6 (27.3)
Medium	3	1 (33.3)	11	1 (9.1)	9	1 (11.1)	23	3 (13.1)
Good	1	0	3	1 (33.3)	6	1 (16.7)	10	2 (20)

## 4 Discussion

The overall prevalence of lungworm infection in the present study was 41% (170/415), with the highest prevalence observed in donkeys (51.3%) and the lowest in mules (20%). These findings are considerably higher than those reported in previous studies. For instance, Getahun (24) documented an overall prevalence of 20% in Bale, while Shiferaw (25) reported 23.2% in Wonchi, Ethiopia. Similarly, Feye and Bekele (26) reported 11.2% in southwest Ethiopia, and Negasa et al. (6) observed 20.1% in the Ambo district of the Oromia region, Ethiopia. The discrepancies between these studies and the present findings may be attributed to variations in environmental conditions, sample sizes, timing of sample collection, and management practices, all of which can influence the survival and transmission dynamics of the parasite larvae Tihitna (27).

In the present study, donkeys exhibited a relatively higher overall prevalence of *D. arnfieldi* infection (51.3%) compared to mules and horses. This finding is consistent with the observations of Legese (28), who reported a prevalence ranging between 30.5 and 32.8% in Dire Dawa and Eastern Ethiopia, as well as with the results of Tihitna Solomon et al. (27), who documented a prevalence of 35.3% in and around Jimma town. The higher infection rate recorded in donkeys can be explained by their role as the primary reservoir host for lungworms, which facilitates continuous transmission of the parasite to other equines sharing the same environment (10). Moreover, this elevated prevalence may also be linked to the comparatively limited veterinary care and management attention given to donkeys despite their heavy workload and significant contribution to transportation and agricultural activities. Such neglect increases their susceptibility to parasitic infections and supports the persistence of lungworm infection within donkey populations (29–31). Moreover, the highest prevalence by locality was recorded in Kafr El-sheikh. The higher humidity and mild temperatures in Kafr El-Sheikh and Qalyubia create favorable conditions for the survival and development of infective larvae on pastures, thereby increasing the risk of infection. In contrast, the drier conditions of Giza may limit larval survival outdoors, but irrigated agricultural areas and shaded animal shelters may still provide microenvironments suitable for transmission. Thus, the interaction between climate, environmental conditions, and equine management practices in these governorates plays a critical role in shaping the epidemiology of lungworm infection (32).

The present study demonstrated that age was a significant determinant in the variation of lungworm infection prevalence among equines (*p* < 0.0001). The infection rate increased with age, being 28.7% in animals younger than 3 years, 39.8% in those aged between 3 and 8 years, and reaching its highest prevalence of 54.7% in animals older than 8 years. These findings are consistent with the results reported by Tihitna Solomon et al. (27), who observed prevalence rates of 18, 10.98, and 22.95% in young, adult, and old animals, respectively. The higher infection rate in older equines could be attributed to prolonged exposure to infective larvae over time, which increases the likelihood of acquiring and harboring the parasite. On the other hand, younger animals, particularly those under 3 years, may also exhibit heightened susceptibility due to their underdeveloped immune systems and limited acquired resistance (27, 33, 34). This aligns with the observations of Feye and Bekele (26), who highlighted that both older and younger age groups tend to have reduced immunity, making them less capable of effectively combating parasitic infections. Additionally, the role of stress, nutritional status, and management practices may further contribute to this age-related difference in prevalence (35, 36).

Consequently, both younger and older equines are considered more susceptible to lungworm infection, as their immune defenses are comparatively weaker, limiting their ability to effectively combat infections (27, 37).

The prevalence of lungworm infection showed seasonal variation; however, the differences were not statistically significant (*p* = 0.838). The highest infection rate was observed during the winter season (52.4%), whereas the lowest prevalence was recorded during the summer (38.2%). The relatively higher occurrence in winter may be attributed to environmental conditions such as lower temperatures and increased humidity, which can enhance the survival and transmission of infective larvae in the environment. In contrast, the lower prevalence observed during summer may be related to high temperatures and dry conditions that reduce larval survival and development (38, 39).

In the current study, body condition was identified as an important factor influencing the prevalence of lungworm infection in equines. The highest infection rate was observed in animals with poor body condition (52.4%), followed by those with medium (38.5%) and good (30.8%) body condition scores. These findings are in close agreement with the results of Tihitna Solomon et al. (27), who reported prevalence rates of 50, 16.3, and 5.2% in animals with poor, medium, and good body condition, respectively. The higher susceptibility of poorly conditioned horses to lungworm infection is likely attributable to inadequate nutrition, which impairs immune function and diminishes the host’s capacity to mount effective defenses against parasitic invasion and clearance (40, 41).

In contrast, equines with medium or good body condition demonstrated comparatively lower infection rates. This may be attributed to their better nutritional state, which supports a stronger immune response and enhances resistance to infection. Additionally, as suggested by Tolossa and Ashenafi (42), animals in better body condition are more frequently engaged in labor activities such as transport or farm work during daytime. Consequently, they spend fewer hours grazing pastures, which decreases their exposure to infective larvae on contaminated grass.

The deworming history of animals was found to be a significant risk factor influencing the prevalence of *D. arnfieldi* infection, with statistical analysis showing a highly significant variation (*p* < 0.0001). A markedly higher prevalence (54.6%) was recorded in equines that had no history of deworming compared to only 18.1% in those that had received anthelmintic treatment. This finding underscores the protective effect of deworming, although the persistence of infection in some treated animals suggests several possible explanations. One reason may be that the commonly used anthelmintics in the area temporarily suppress egg production by adult worms rather than eliminating them completely. Alternatively, resistance of the parasite to the available drugs could be emerging, or the poor quality of some locally used anthelmintic preparations might limit their efficacy (33, 43).

Interestingly, despite the absence of treatment, 46.8% of non-dewormed animals were not infected with lungworms. This could be attributed to the development of acquired immunity following repeated prior exposures, which helps to limit reinfection (7, 44). Another explanation may be the lack of recent contact with infective larvae in their grazing environment. Moreover, at the time of sampling, some animals could have harbored infections that were not yet patent; since the prepatent period of *D. arnfieldi* is approximately 4 weeks, larvae may have been present in the lungs without egg shedding. In addition, larvae can undergo hypobiosis (dormancy) for up to 5 months under unfavorable conditions, thereby preventing detection during routine fecal examination. Despite providing valuable insights, this study was limited by its relatively small sample size and geographic coverage, which may not fully represent the national epidemiological situation. Additionally, molecular identification of *D. arnfieldi* was not performed which could have enhanced the diagnostic accuracy and allowed for the detection of subclinical or mixed infections.

This study has certain limitations that should be acknowledged. Morphological and histopathological examinations were not performed, as the primary objective was to investigate the prevalence and associated risk factors of *D. arnfieldi* infection under field conditions. Consequently, the absence of these analyses limited our ability to correlate parasitological findings with tissue-level pathological alterations. Future studies should incorporate detailed morphological characterization and histopathological evaluation to provide a more comprehensive understanding of the pathological effects of *D. arnfieldi* infection in equines.

## 5 Conclusion

The current study demonstrated a notably high prevalence of *D. arnfieldi* infection (41%) among equines in Egypt, with donkeys exhibiting the highest rate of infection, confirming their crucial role as the main reservoir host for lungworms. The results highlighted that factors such as age, species, body condition, and deworming history significantly influenced infection risk, with older, poorly conditioned, and non-dewormed animals being more vulnerable. These findings emphasize the importance of adopting enhanced management strategies, implementing routine and efficient deworming programs, and providing focused veterinary care—particularly for donkeys—to minimize the impact of lungworm infections and promote better health and productivity in equine populations. Although the observed prevalence may raise concerns regarding the effectiveness of current control measures, the possible roles of anthelmintic resistance or drug quality cannot be determined from the present data and warrant further investigation in future studies.

## Data Availability

The original contributions presented in the study are included in the article/supplementary material, further inquiries can be directed to the corresponding author.

## References

[1] Elsawy BS Mahmoud MS Suarez CE Alzan HF. Impact of equine and camel piroplasmosis in Egypt: how much do we know about the current situation? Pathogens. (2023) 12:1318. doi: 10.3390/pathogens12111318, 38003783 PMC10675018

[2] Sapakota C. (2009) A Report on Prevalence of Helminthes Parasites in Mules of Brick Kiln.

[3] Esch KJ Petersen CA. Transmission and epidemiology of zoonotic protozoal diseases of companion animals. Clin Microbiol Rev. (2013) 26:58–85. doi: 10.1128/CMR.00067-12, 23297259 PMC3553666

[4] Mulate B. Preliminary study on helminthosis of equines in south and north Wollo zones. J Vet Assoc. (2005) 9:25–37.

[5] Abd Elmohsen M Selim A Abd Elmoneim AE. Prevalence and molecular characterization of lumpy skin disease in cattle during period 2016-2017. Benha Vet Med J. (2019) 37:173–6. doi: 10.21608/bvmj.2019.18293.1118

[6] Negasa T Dilbato T Gudeta D. Cross-sectional study on equine lung worm and associated risk factor in ambo district, Oromia region, Ethiopoia. Int J Res Granthaalayah. (2017) 5:312–9. doi: 10.5281/zenodo.802336

[7] Urquhart G.M. (1996) Veterinary Parasitology 2nd edition, Blackwell Ltd, Oxford, 224–234.

[8] Smith BP. Large Animal Internal Medicine-E-Book: Large Animal Internal Medicine-E-Book.Elsevier Health Sciences (2014).

[9] Barrandeguy ME Carossino M. Infectious diseases in donkeys and mules: an overview and update. J Equine Vet Sci. (2018) 65:98–105. doi: 10.1016/j.jevs.2018.02.026

[10] Rose R.J. Hodgson D.R. (1993). Equine Veterinary Education, 5:221–221.

[11] Bowman D. (1999). Georgis' Parasitology for Veterinarians. Philadelphia: W.B. Saunders Company.

[12] Mandal S. "Veterinary nematology". In: Textbook of Veterinary Parasitology Springer (2025). p. 221–384.

[13] Junquera P. "Dictyocaulus species, parasitic lungworms of cattle, sheep, goats and other livestock. Biology, prevention and control. Dictyocaulus filaria, Dictyocaulus viviparus, Dictyocaulus arnfieldi". In: Merck Veterinary Manual. Whitehouse Station: Merck and Co, Inc (2014). p. 14–28.

[14] Patel R Chauhan T Srivastava S Mishra P Jain P. "Parasitism in farm animals". In: Prospects of Fungal Biotechnologies for Livestock Volume 1: Fungal Bioengineering in Livestock Health Management (2025). p. 1–42. doi: 10.1016/j.jevs.2023.104915

[15] Abera G. Prevalence of Lungworm Infection and Associated Risk Factors among Sheep with Respiratory Signs and Evaluation of Public Intervention for Control and Prevention in North Shewa Zone, Oromia, Ethiopia.Haramaya University (2022).

[16] Fraser C.M. (1991). The Merck Veterinary Manual: A Handbook of Diagnosis, Therapy, and Disease Prevention and Control for the Veterinarian.

[17] Saadi A Tavassoli M Dalir-Naghadeh B Samiei AJ. A survey of *Dictyocaulus arnfieldi* (Nematoda) infections in equids in Urmia region, Iran (2018) 64:235–240. 10.17420/ap6403.158 30316220

[18] Sazmand A Bahari A Papi S Otranto DJ. Parasitic diseases of equids in Iran (1931–2020): a literature review. Parasit Vectors. (2020) 13:586. doi: 10.1186/s13071-020-04472-w, 33213507 PMC7676409

[19] Thrusfield M. Veterinary Epidemiology. Wiley-Blackwell: John Wiley & Sons, 896. (2018).

[20] Foreyt WJ. Veterinary Parasitology Reference Manual. John Wiley & Sons (5th edition), 256. (2013).

[21] Cameron TW. On the morphology of the adults and the free living larvae of *Dictyocaulus arnfieldi*, the lung-worm of equines. J Helminthol. (1926) 4:61–8.

[22] Seyoum Z Tesfaye M Derso S. Prevalence, intensity and risk factors of infestation with major gastrointestinal nematodes in equines in and around Shashemane, southern Ethiopia. Trop Anim Health Prod. (2015) 47:1515–21. doi: 10.1007/s11250-015-0893-5, 26205906

[23] Pyrek P Siwinska N Zak-Bochenek A. Reproducibility of the body condition score assessment in Silesian horses, using the 9-point BCS scale. Vet Res Commun. (2023) 47:273–8. doi: 10.1007/s11259-022-09916-5, 35316481

[24] Getahun F. (1993) Helminths and External Parasites of Equines Species in Bale Administrative Region, Ehiopia.

[25] Shiferaw Y. (1993) "Preliminary Survey on Heliminthosis and Management Practice of equine in Wenchi Awraga, Ethiopia. doi: 10.5281/zenodo.802336

[26] Feye A Bekele T. Prevalence of equine lung worm (*Dictyocaulus arnfieldi*) and its associated risk factors in Jimma town, south West Ethiopia. Adv Life Sci Technol. (2016) 40:26–30.

[27] Solomon T Bogale B Chanie M Melaku A. Occurrence of lungworm infection in equines and their associated risk factors. Glob Vet. (2012) 8:35–8.

[28] Legese Y. (1996) Preliminary Survey on Management Aspects and Health problem of Donkey in Dire Dawa and East Oromia, Ethiopia.

[29] Selim A Abdelhady A. Neosporosis among Egyptian camels and its associated risk factors. Trop Anim Health Prod. (2020) 52:3381–5. doi: 10.1007/s11250-020-02370-y, 32929587

[30] Selim A El-Haig M Galila ES Gaede W. Direct detection of *Mycobacterium avium* subsp. *Paratuberculosis* in bovine milk by multiplex real-time PCR. Anim Sci Paper Rep. (2013) 31:291–302.

[31] Tesfaye A Curran MM. A longitudinal survey of market donkeys in Ethiopia. Trop Anim Health Prod. (2005) 37:87–100. doi: 10.1007/s11250-005-9010-5, 16335073

[32] Selim A Khater HJCI. Seroprevalence and risk factors associated with equine piroplasmosis in North Egypt. Microbiol Dis. (2020) 73:101549. doi: 10.1016/j.cimid.2020.101549 32950955

[33] Abdulkadir K Ibrahim N Deneke Y. Prevalence of equine lungworm and associated risk factors in Sudie district, Oromia region, south eastern Ethiopia. Afr J Agric Res. (2017) 12:1526–31. doi: 10.5897/AJAR2016.11683

[34] Selim A.M. Elhaig M.M. Gaede W. (2014) Development of Multiplex Real-Time PCR Assay for the Detection of *Brucella* Spp., *Leptospira* Spp. and *Campylobacter Foetus*. 10.12834/VetIt.222.702.3 25546064

[35] Selim A Abdelrahman A Thiéry R Sidi-Boumedine K. Molecular typing of *Coxiella burnetii* from sheep in Egypt. Comp Immunol Microbiol Infect Dis. (2019) 67:101353. doi: 10.1016/j.cimid.2019.101353, 31605891

[36] Selim A Attia KA Alsubki RA Kimiko I Sayed-Ahmed MZ. Cross-sectional survey on *Mycobacterium avium* Subsp. paratuberculosis in dromedary camels: Seroprevalence and risk factors. Acta Trop. (2022) 226:106261. doi: 10.1016/j.actatropica.2021.106261, 34848184

[37] Alshammari A Gattan HS Marzok M Salem M Al-Jabr OA Selim AJ. *Fasciola hepatica* infection in horses in three governorates in northern Egypt: prevalence and risk factors. J Equine Vet Sci. (2023) 130:104915 37652146 10.1016/j.jevs.2023.104915

[38] Alshammari A Gattan HS Marzok M Selim AJSR. Seroprevalence and risk factors for *Neospora* spp. infection in equine in Egypt. Sci Rep. (2023) 13:20242. doi: 10.1038/s41598-023-47601-y 37981658 PMC10658168

[39] Selim A Shoulah S Abdelhady A Alouffi A Alraey Y Al-Salem WSJVS. Seroprevalence and risk factors associated with canine leishmaniasis in Egypt. Vet Sci. (2021) 8:236. doi: 10.3390/vetsci8100236 34679066 PMC8541007

[40] Fekete SG Kellems R. Interrelationship of feeding with immunity and parasitic infection: a review. Vet Med. (2007) 52:131–43. doi: 10.17221/2028-vetmed

[41] Weatherhead JE Gazzinelli-Guimaraes P Knight JM Fujiwara R Hotez PJ Bottazzi ME . Host immunity and inflammation to pulmonary helminth infections. Front Immunol. (2020) 11:594520. doi: 10.3389/fimmu.2020.594520, 33193446 PMC7606285

[42] Tolossa Y Ashenafi H. Epidemiological study on gastrointestinal helminths of horses in Arsi-bale highlands of Oromiya region, Ethiopia. Ethiop Vet J. (2013) 17:51–62. doi: 10.4314/evj.v17i2.4

[43] Selim A Weir W Khater HJVW. Prevalence and risk factors associated with tropical theileriosis in Egyptian dairy cattle. Vet World. (2022) 15:919 35698515 10.14202/vetworld.2022.919-924 PMC9178591

[44] Radostits OM Blood DC Gay CC (1997). Veterinary Medicine: A Textbook of the Diseases of Cattle, Sheep, Pigs, Goats and Horses. Elsevier.

[45] Tihitna Solomon TS Basaznew Bogale BB Mersha Chanie MC Achenef Melaku A.M. Ocuurrence of Lungworm Infection in Equines and their Associated Risk Factors. *Global Veterinaria*, (2012) 8:35–38.

[46] Thienpont D Rochette F Vanparljs OFJ. (1986) Diagnosing Helminthiasis by Coprological Examination. Janssen Reseach Foundation, Beerse Belgium. (1986).

[47] Kiszely S Gyurkovszky M Solymosi N Farkas R. Survey of lungworm infection of domestic cats in Hungary. Acta Veterinaria Hungarica, (2019) 67:407–417. 31549542 10.1556/004.2019.041

